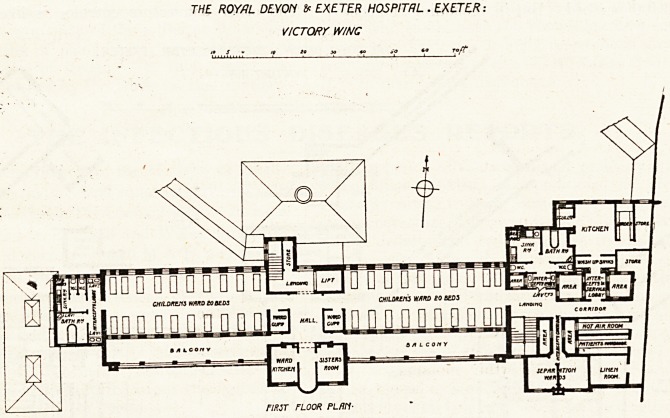# Royal Devon and Exeter Hospital

**Published:** 1920-09-18

**Authors:** 


### HOSPITAL ARCHITECTURE AND CONSTRUCTION.

### Royal Devon and Exeter Hospital.

### Victory Wing Addition.

We are publishing plans which have been furnished by the architects, Messrs. E. H. Harbottle & Sons, of an addition—known as the Victory Wing—to the Royal Devon and Exeter Hospital. We believe that the work of construction is now in progress and that completion is expected some time in 1921. The contemplated expenditure is thirty thousand pounds, and the work has been entrusted to Mr. E. C. Lee of Exeter as contractor. There will be seventy-eight beds in this addition (of which forty are for children), so that the cost works out at under £400 per bed. But in reckoning the cost floor and the first floor, and some ingenuity is displayed in so planning the main staircase as to receive the corridor at an intermediate landing. But if, as would appear to be the case, the level of the corridor floor is at least five feet above the ground floor, and its roof some eight to ten feet higher, it is clear that the north windows at the east end of the western ward must be rather badly obscured by the corridor building. It is probable, however, that the plan is incorrect at this point, or has suffered by undue reduction.

The other connecting corridor is likely, in our per bed of an addition to a hospital it must, of course, always be borne in mind that the addition has a share in general and administrative parts of the original building which do not form a part of the new buildings. It is clear from indications on the plans that the disposition of the new structure has been largely influenced by the existing buildings and by the necessary connections thereto; but it is a matter of regret that the plans supplied do not give fuller information on this point. It will be seen that there are two points of contact, one by means of an oblique corridor surmounting an arcade which strikes the new buildings at the central hall; the other by a corridor which flanks the new wing along its eastern end.

The former corridor appears to reach the new block at a level intermediate between the ground opinion, to be unduly dark, for it relies at its centre on a window in an area which scales only seven feet across, and it is the mention of this area which brings us to the quality which marks the defect of this otherwise straightforward and businesslike design. It is true that some modern hospital architects, and in particular our American rivals, are abandoning faith in the "cut-off" principle. The intercepting lobby is in fact losing favour in some quarters, being driven away by the unyielding force of economy. But economy at its bravest is apt to become parsimony, and in these columns we have continued to plead for the maintenance of that system of isolation by air current which has been from the days of Florence Nightingale onwards one of the hall-marks of English hospital sanitation.

**Hospital Architecture**—(*continued*).

The architects in this case bow to the letter of the law, but are ungenerous to the spirit. We do not fail to realise that in all probability Messrs. Harbottle were driven by circumstances to do what they have done, and that they would be willing to agree with us in regretting the result. The fact, as will be seen from the plans, is that the very existence of cross-ventilation in this design hangs precariously upon the provision of a couple of enclosed areas, one of which scales eight by eight and the other five by five. As the latter area or shaft is occupied on one of its sides by the sole window of a w.c., it is clear that its powers of supplying a current of pure air are only nominal.

It is possible, of course, that provision is made, though not shown on the plan, for admitting air to the base of these small areas (there are five in all), but the general impression produced by this part of the plan—the block at the east end—is that the architects have rather too blind a confidence in areas, whatever their size or lack of size may be.

The designers, in a written description, explain that the wards of the ground floor measure fifty-six by twenty-eight feet each, and together accommodate forty patients. But it will be seen from the plan that other counsels have prevailed. A space having been cut off the end of each ward to provide single rooms (and dark rooms), the remaining length is about forty-five feet. The architects have accordingly cut down the accommodation in each main ward to sixteen beds. This arrangement gives a floor area per bed of about eighty square feet, which is quite little enough.

The surgeons' room attached to the operating-theatre is a useful feature (too often forgotten or crowded out), and the general arrangement of the theatre appears good, though the window lighting is probably shown incorrectly on the plans.

It would be interesting to know what is the general kitchen system of the hospital. The kitchens —each about sixteen feet square, one on each floor, and distinct from the "ward kitchens"—would appear to indicate a departmental arrangement which must require a large staff.

Our general opinion of the scheme as exhibited by the plans here published, is that the architects have shown great ingenuity in packing accommodation on to a limited space, and that this ingenuity has here and there been practised at the expense of considerations which, except under severe stress of circumstances, ought not to be too lightly set aside.

THE ROYAL DEVON & EXETER HOSPITAL. EXETER:

VICTORY WING

FIRST FLOOR PLAN

## Figures and Tables

**Figure f1:**
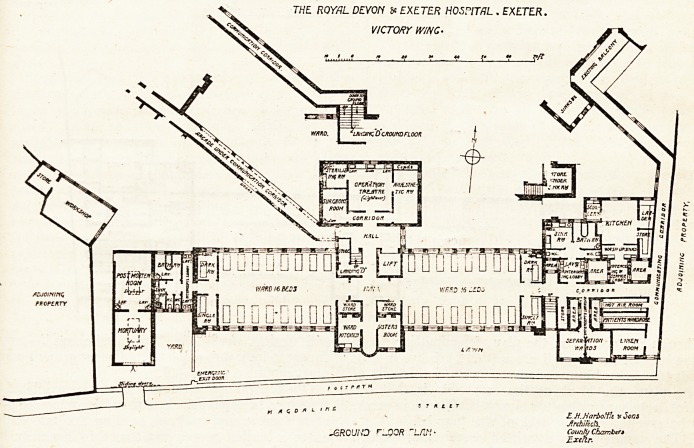


**Figure f2:**